# Understanding Discourse in Face-to-Face Settings: The Impact of Multimodal Cues and Listening Conditions

**DOI:** 10.1037/xlm0001399

**Published:** 2024-10-14

**Authors:** Anna Krason, Rosemary Varley, Gabriella Vigliocco

**Affiliations:** 1 Department of Experimental Psychology, University College London; 2 Department of Language and Cognition, University College London

**Keywords:** multimodal communication, language comprehension, gestures, mouth movements, background noise

## Abstract

In face-to-face contexts, discourse is accompanied by various cues, like gestures and mouth movements. Here, we asked whether the presence of gestures and mouth movements benefits discourse comprehension under clear and challenging listening conditions and, if so, whether this multimodal benefit depends on the communicative environment in which interlocutors are situated. In two online experiments, participants watched videoclips of a speaker telling stories, and they answered yes–no questions about the content of each story. The speaker in the videos was spontaneously gesturing (or kept her hands still) and was wearing a surgical mask (or had her lips visible). The experiments differed in the communicative environment. In Experiment 1, the speaker narrated stories in silence, whereas the listener (participants) heard them in clear or degraded speech conditions (analogous to watching the news on TV in a quiet or noisy café). In Experiment 2, the speaker narrated the stories once in silence and once while listening to background noise, and the listener heard them in clear or degraded speech condition, respectively (analogous to listening to a friend in a quiet or noisy café). Across the experiments, we found that cospeech gestures facilitated discourse comprehension regardless of the listening conditions or the presence of a mask. In contrast, mouth movements were primarily helpful in challenging listening conditions. These findings indicate that both cues matter to listeners but to a different extent. Moreover, we found that the multimodal benefit to comprehension was similar regardless of the communicative environment. Thus, this study demonstrates the importance of both cospeech gestures and mouth movements to discourse comprehension, offering insights into the dynamic interplay between these cues under different communicative environments.

Discourse refers to a linguistic unit larger than a sentence and that often describes a connected set of events. It is generally agreed that listeners process discourse by building situation models of the described events using general knowledge, communicative context, and prior experiences (Zwaan, Langston, et al., 1995; Zwaan, Magliano, et al., 1995; Zwaan & Radvansky, 1998). Studies investigating discourse comprehension have primarily centered around the linguistic information conveyed by speech or text. However, in face-to-face settings, spoken discourse is accompanied by an abundance of communicative cues, such as hand gestures, mouth and facial movements, body posture, eye gaze, and prosody, that support the creation of situation models and, in turn, impact discourse comprehension (Clark, 1996; Goodwin, 1981; Kendon, 1980, 2004; McNeill, 1992; Schegloff, 1984). Moreover, the importance of visual cues became particularly prominent during the COVID-19 pandemic because wearing health masks muffles speech and hides important mouth and facial movements, while at the same time it might drive listeners to rely upon other available cues, like gestures. It is still unclear to what extent these, often referred to as “nonlinguistic” cues, improve comprehension as most studies have investigated isolated words accompanied by either gestures or mouth movements, ignoring the possibility that these cues may interact with each other and with the auditory signal. The aim of the present study was to fill this gap by looking at how spontaneously produced cospeech gestures and wearing a face mask impact discourse comprehension under clear and challenging listening conditions.

## Cospeech Gestures in Comprehension

Cospeech gestures (or gesticulations; Kendon, 1988) are spontaneous and idiosyncratic hand movements that are time-locked to speech. They are ubiquitous in face-to-face discourse, and studies have shown that they serve a communicative function (Hostetter, 2011; Kendon, 2004). Representational gestures, which refer to physical or abstract features and properties of objects, actions, and elements of events in an imagistic way (e.g., rapidly moving index and middle fingers to represent a walking action or pointing behind oneself to refer to past times), facilitate discourse comprehension by activating the semantic system (Kendon, 1988; Kita et al., 1997; McNeill, 1992). These gestures are particularly beneficial when the information they convey is spatial or motoric (e.g., describing directions), which has been explained in terms of a construction of a spatially organized situation model (Cutica & Bucciarelli, 2013). It has been suggested that representational gestures are integrated with speech during online comprehension, as they are automatically processed even when the semantic information they convey mismatches the speech (Green et al., 2009; Kelly et al., 2010; McNeill et al., 1994). In line with Kintsch’s model of comprehension (Kintsch, 1998; Kintsch & van Dijk, 1978; van Dijk & Kintsch, 1983), representational gestures could be integrated both at the semantic and situation model levels, thus supporting interpretation of the discourse.

While many studies have investigated the representational gestures that speakers produce while narrating (e.g., Alibali et al., 2000; A. Brown & Gullberg, 2008; Kita & Özyürek, 2003; McNeill & Levy, 1982), only a few have looked at the role of these gestures in discourse comprehension. In three studies, Dargue and Sweller (2018, 2020) and Dargue et al. (2019) presented participants with videos of a speaker narrating short stories and asked them to recall as much information as possible. On half of the occasions, the speaker also produced iconic (representational) gestures that were either “typical” (most frequently occurring gestures obtained from a norming retell task) or “atypical” (a less common form of gestures created by the experimenters). A general conclusion from these studies was that typical, but not atypical, gestures improve free recall performance, which was further explained in terms of greater semantic relatedness between the information provided by typical gestures and speech (Dargue & Sweller, 2018, 2020; Dargue et al., 2019). Similar findings were reported by McKern et al. (2021), who used the same set of materials but additionally manipulated the clarity of the audio using background noise to increase task difficulty. Again, only typical gestures were found to facilitate narrative comprehension, and this effect was particularly large in people with poorer nonverbal memory, as measured with a delayed recall task. The authors interpreted this finding in terms of typical gestures being most beneficial during semantic processing because they leave a long-term memory trace, thanks to their iconicity. There was no interaction with speech clarity, suggesting that commonly occurring iconic gestures benefit speech comprehension regardless of task difficulty. However, the lack of effect of atypical iconic gestures can be related to the fact that they were generated artificially; therefore, they might have introduced a mismatch with the auditory information, which led to poorer performance relative to when typical gestures accompanied speech.

Although some have argued that discourse comprehension is primarily influenced by representational gestures because they support the creation of situation models by bringing events to interlocutors’ eyes (Feyereisen, 2006; Murgiano et al., 2021), other types of gestures may also play a role. These include beat gestures, which are rhythmic movements tightly coupled with speech prosody that mark salient phrases or words (Krahmer & Swerts, 2007) and have been found to elicit enhanced activation in the auditory cortex (Hubbard et al., 2009); pragmatic gestures, such as moving an open, flat hand toward the listener, which serve an interactive function (e.g., turn taking; Bavelas et al., 1992, 1995); and deictic gestures, which include abstract pointing gestures indicating the location of a physically absent referent (e.g., pointing to a particular location in space to refer to the same protagonist in a story; Kendon, 1988, 2004; McNeill, 1992) and have been found to support discourse cohesion. For instance, Gunter et al. (2015) used electroencephalography to measure the impact of abstract pointing gestures that indicated the location of the protagonists described in a story that was either congruent (i.e., consistent with previously established location) or incongruent (i.e., inconsistent with previously established location). Larger negative component peaking around 400 ms after the stimulus and positive component peaking around 600 ms after the stimulus effects, which are event-related potentials often associated with language processing difficulty (Kutas & Federmeier, 2011), were found for the incongruent gestures, suggesting that listeners automatically process these gestures during comprehension, similarly to the iconic ones (e.g., Kelly et al., 2010). Furthermore, deictic gestures can reactivate situational models even when gestures are not in view anymore (Sekine & Kita, 2017) or when sentence meaning is ambiguous (Goodrich Smith & Hudson Kam, 2012; Sekine & Kita, 2015).

To summarize, previous research has shown that representational and deictic gestures support discourse comprehension, thanks to the semantic relatedness between gesture and speech information. Less is known about the extent to which observing other types of gestures, such as beat or pragmatic gestures, benefits discourse comprehension and whether any such benefit is influenced by gesture frequency or kinematics (e.g., amplitude). Moreover, in all the studies described above, the gestures were scripted rather than naturally occurring, which may introduce differences in processing. Our study contributes to this line of investigation by looking at nonscripted and spontaneous gestures (including representational, deictic, pragmatic, and beat gestures).

## Visual Speech Cues and the Effect of Masks

Face and mouth movements are also central cues for speech comprehension. Mouth movements, in particular, tap onto the phonological level of processing and have been shown to aid speech recognition by constraining lexical competition (Lachs & Pisoni, 2004; Peelle & Sommers, 2015; Tye-Murray et al., 2007). Most of what we know about the role of mouth movements in speech comprehension comes, however, from studies investigating isolated words (or embedded in sentences), while less is known about their impact on spoken discourse comprehension. Using a shadowing task, Reisberg et al. (1987) demonstrated that the presence of mouth movements speeds up the processing of both clear and distorted spoken passages. A facilitatory effect of mouth movements was also reported for accuracy performance. Arnold and Hill (2001) presented short passages in auditory-only and audiovisual modalities followed by comprehension questions. The passages were either presented in listeners’ native versus nonnative language, accompanied by different accents, and semantically/syntactically complex versus simple sentences. The results showed that comprehension of narratives was always better when mouth movements were visible, suggesting that visual speech information is automatically processed during comprehension of passages.

The interest in the role of mouth movements has grown in the last couple of years because the COVID-19 pandemic has forced people to wear face masks covering mouth and cheeks and making speech muffled. Studies investigating the effect of face masks on speech intelligibility have demonstrated that both cognitive and listening effort increase (Giovanelli et al., 2021), which is particularly detrimental for people with hearing impairment (Saunders et al., 2021) and cochlear implants (Homans & Vroegop, 2021). In addition, emotional processing is hindered (Mheidly et al., 2020), which further leads to decreased trust, particularly in clinical populations (Malik et al., 2021). Interestingly, people are still quite accurate at processing emotions even when the mouth is covered with a mask provided the rest of the body is visible (P. Ross & George, 2022). This finding suggests that interlocutors flexibly weight the incoming information, and if one cue is unavailable (or less informative), they make use of other cues (Krason et al., 2021; Skipper et al., 2009; Zhang, Frassinelli, et al., 2021).

Masks can be of different sizes, shapes, and materials; however, this does not appear to affect speech intelligibility as shown in work by V. A. Brown et al. (2021). The authors tested a group of younger and older adults on a comprehension task with sentences embedded in noise. The participants’ task was to watch videoclips of a speaker producing sentences and type down what they had heard. The speaker in the videos was asked to wear different health masks (surgical, cloth with filter, cloth without filter, transparent, or no mask). The results show that wearing a mask (regardless of its type) hinders sentence identification in noise in both younger and older adults to a similar extent. This finding is in line with studies demonstrating that mouth movements are particularly beneficial in noisy listening conditions (Grant & Seitz, 2000; Ma et al., 2009; L. A. Ross et al., 2007; Schwartz et al., 2004; Sumby & Pollack, 1954).

Finally, Pycha et al. (2022) investigated auditory sentence comprehension under various background noise conditions using a final word identification task. They manipulated factors such as whether the speaker was wearing a face mask (vs. not), speaking style (clear or casual, i.e., when a speaker was asked to produce speech in a natural way), and speaker information (image of a person with or without a mask). Across two experiments, the authors showed that speech intelligibility was higher when the speaker was wearing a mask, which was interpreted in terms of the Lombard effect (Junqua, 1993), such that the speaker adjusted their voice to compensate for the presence of the mask, making the speech more intelligible. Comprehension also improved when the speech was clear rather than casual and when listeners knew that the speaker was wearing a mask. These results demonstrate that speech comprehension is a dynamic social act involving speaker–listener interactions, which depend on the information that interlocutors have about each other (see also Trujillo et al., 2021).

In brief, mouth and facial movements facilitate speech processing, but their effect becomes more prominent when the auditory encoding is challenging. For example, observing an interlocutor’s mouth movements in noisy listening conditions can help disambiguate what is being said. Moreover, while wearing a health mask covering important facial cues became a standard during the COVID-19 pandemic, it is not clear whether, and if so, masks affect discourse comprehension. Finally, as with cospeech gestures, little is known whether the benefit of mouth movements depends on their interactions with other available cues.

## Interactions of Hand and Mouth Movements

Traditionally, different cues such as gestures and mouth movements have been studied in isolation, thus neglecting the possibility that cues may interact during speech comprehension and production. Only recently, the co-occurrence of multiple cues alongside speech has been investigated, with a handful of studies looking at connected speech (Trujillo et al., 2021; Zhang, Frassinelli, et al., 2021). For example, in a series of electroencephalography experiments, Zhang, Frassinelli, et al. (2021) presented participants videos of a speaker uttering short passages while spontaneously producing gestures (including representational and beat gestures). The speaker’s mouth movements were always visible, as it is most often the case in naturalistic settings. The authors quantified linguistic information (word predictability), as well as the information contained in speech (prosody), and visual cues (gestures and mouth movements) and measured oscillatory changes in the negative component peaking around 400 ms after the stimulus event-related potential. The results show that multimodal cues always modulate the comprehension of spoken passages, but they do so in an interactive and dynamic way. For example, mouth movements become particularly useful when gestures are also present, suggesting a multimodal enhancement to comprehension.

Trujillo et al. (2021) asked whether the Lombard effect is modulated by multimodal (including gesture and mouth) cues. The authors investigated face-to-face dyadic interactions, during which participants wore headphones and listened to a multitalker babble. One of the participants had to communicate (in any manner they chose) action-related verbs to their experimental partner, whose task was to correctly identify these verbs. The results demonstrate that speakers adjust all multimodal cues when faced with their listeners in adverse listening conditions, suggesting that the Lombard effect is a multimodal, rather than an auditory-only, phenomenon and that these modulations are helpful for both interlocutors. In addition, gestures interact with speech such that louder voice was reported particularly in the absence of gestures, whereas more exaggerated gestures were observed when the voice was neutral. Thus, gestures can be used as an additional channel to overcome noise. Modulations of mouth movements were only found in some participants, suggesting individual differences.

Other studies have further demonstrated that the communicative environment the interlocutors are situated in, that is, whether a speaker/listener is in a noisy or quiet environment, matters to language comprehension. For example, Garnier et al. (2018; see also Garnier et al., 2010) investigated speech intelligibility in clear and degraded conditions across three communicative scenarios: interactive audiovisual, in which the speaker interacts with the listener face-to-face; auditory-only; and reading aloud. The results demonstrated that visual facial cues (just as acoustics) are modulated in the presence of background noise, but crucially this effect was contingent upon the visibility of the listener to the speaker. Overall, these findings are in line with Pycha et al. (2022) and Trujillo et al. (2021) and suggest context-dependent modulations where audiovisual adjustments to noise are at least partially made for the listener.

Two important conclusions can be drawn from the above review. First, visual cues modulate linguistic information, and they do so in a manner that depends on the informativeness of other cues. Second, speakers automatically adjust the way they produce visual cues in adverse listening conditions (multimodal Lombard effect), and these adjustments are suggested to be, at least in part, listener-oriented. It is still unclear, however, whether these cues impact discourse comprehension differently depending on listening and speaking environments. We contrast two face-to-face communicative environments in this study: watching TV news in a café where the news presenter always produces cues in a quiet environment but the listener experiences them in silence (clear speech condition) or in noise (degraded speech condition) versus listening to a friend in a café where the friend and the listener are situated in the same communicative environment (either quiet or noisy).

## The Present Study

In this study, we asked (a) whether and how visual cues, such as cospeech gestures and mouth movements, benefit discourse comprehension under clear and challenging listening conditions and (b) whether producing these visual cues in different communicative environments (i.e., in noise or in silence) impacts comprehension differently. In two separate experiments, we presented participants with videos of an actress telling stories about everyday events or retelling an episode from a Tom and Jerry or Sylvester and Tweety Pie cartoon. Participants were asked to answer eight yes–no comprehension questions about the content of each video story. We manipulated the following: (a) the actress in the videos was either spontaneously gesturing or kept her hands still alongside her body while telling the stories (gesture present vs. absent condition); (b) the face of the actress was fully visible or her lips and cheeks were covered with a surgical mask (mouth present vs. absent condition); and (c) the audio in the videos was clear (unedited version) or background noise was manually added (clear vs. degraded speech condition). The decision to use background noise (rather than any other noise) and a health mask (rather than e.g., blurring the area of the lips) was driven by their seminaturalistic character, as people often need to process discourse in a noisy environment, and wearing a mask has become a standard since the COVID-19 pandemic. The manipulations remained constant across the experiments, but the communicative environment in which the speaker narrated the stories differed between experiments. In Experiment 1, the speaker produced the story in silence, and the listener watched the videos in either silence or noise. In Experiment 2, both the speaker and the listener were in a quiet or a noisy environment. For the degraded (noisy) condition in Experiment 2, the actress was wearing earphones and listening to cafeteria-type noise.

On the basis of prior studies investigating multimodal speech comprehension, we predicted that the presence of visual cues would facilitate comprehension of discourse (Zhang, Frassinelli, et al., 2021), and this effect would be most robust in adverse listening conditions (Drijvers & Özyürek, 2017; Krason et al., 2021). Moreover, we predicted that gestures would benefit comprehenders to a larger extent than mouth movements, given that they impact linguistic processing differently, that is, gestures primarily support semantic processing while mouth movements support phonological processing (Hirata & Kelly, 2010). Additionally, if speakers automatically make multimodal adjustments while narrating in noise (as in the case of when the actress wore earphones and listened to background noise) and if these adjustments are, at least partially, intended for the listener (Garnier et al., 2018; Pycha et al., 2022; Trujillo et al., 2021), it is likely that we will observe a larger advantage of gestures and mouth movements on discourse comprehension in Experiment 2 than in Experiment 1.

The experiments were carried out under University College London Ethical Approval (0143/003) and were preregistered using https://aspredicted.org/. The preregistrations,^1^ data, example materials, and the R code are publicly available and can be found on the Open Science Framework (OSF) at https://osf.io/6zxuw/.

## Experiment 1

Experiment 1 assesses the impact of cospeech gestures, visible mouth movements, and listening conditions on discourse comprehension. In this experiment, the speaker always produced speech and multimodal cues in a quiet environment, while the listener (participants) processed the incoming information in either a quiet or noisy environment (e.g., similarly to watching TV news in a quiet or noisy café).

### Method

#### Participants

We recruited 100 native speakers of American English via Prolific (https://www.prolific.co/). They were all right-handed monolinguals and reported no language, hearing, vision, or neurological impairments. The number of participants was determined using three separate methods. Simulations analysis with *simr* package (Green & MacLeod, 2016) based on coefficients from our previous study investigating interactions between gestures and mouth movements (Krason et al., 2021) suggested that 80 participants would be enough to detect a three-way interaction with almost 95% power at an α level at .05. Using two other sample-size calculations methods, we found that 100 participants would be needed to detect an effect size of 0.3 (GPower analysis; Faul et al., 2009) and have a good-to-excellent level of replicability and good level of precision (Trafimow, 2018; see the preregistration for more details).

Before the analyses, outliers were identified and removed. One individual performed below 3 *SD* from the group mean, and one other had an average reaction time above 3 *SD* from the group mean. The data from the remaining 98 participants (*M*_age_ = 30.33, *SD* = 6.31; 49 females, 48 males, one preferred not to say) were used for the analyses.

#### Materials

The stimuli consisted of 16 short stories, each accompanied by eight yes–no comprehension questions about the content of the stories. Half of the stories and the corresponding questions were taken from the Discourse Comprehension Test (DCT; Brookshire & Nicholas, 1993), which is a narrative comprehension assessment developed for adults with brain damage that has been standardized on neurologically healthy groups. The DCT contains 10 different stories, but only eight were selected for the purpose of this study. The other half of the stories were written by the experimenters and were based on four episodes of Tom and Jerry and four episodes of Sylvester and Tweety Pie cartoons. Cartoon-type stories have been previously used to investigate the use of gestures during storytelling and elicitation tasks (e.g., A. Brown & Chen, 2013; A. Brown & Gullberg, 2008; Gullberg & Kita, 2009; Kita & Özyürek, 2003; McNeill & Levy, 1982). Cartoon stories are also more concrete and involve more actions; thus, we expected to see a larger proportion of representational gestures relative to other types of gestures in these stories. The DCT stories are more abstract and should involve fewer such gestures. The reason for including different types of stories was to ensure variability and to minimize the effects of fatigue, boredom, and/or story familiarity. The DCT and cartoon stories were matched as closely as possible by the number of words, number of unfamiliar words (i.e., words that fall outside of the classification of the 10,000 most frequently used words; Carroll et al., 1971), number of sentences, mean sentence length (average number of words per sentence), number of subordinate clauses, and listening difficulty (i.e., the average number of syllables that are more than one per word for each sentence, averaged across sentences per story; the Easy Listening Formula from Fang, 1966). The questions for the cartoon stories were also modeled on those of the DCT, focusing on information salience (questions about main ideas that were central to the stories vs. details that were not crucial to understanding the message of the stories) and directness (questions about the information directly stated in the stories vs. implied information that could be inferred). Thus, there were four types of questions: main idea-stated, main idea-implied, detail-stated, and detail-implied, and each type appeared twice per story: once with the correct “yes” response and once with the correct “no” response. See Table 1 for the comparison between DCT and cartoon stories, with example stories and questions. Full materials are available on the project’s OSF at https://osf.io/6zxuw/.[Table tbl1]

**Figure 1 fig1:**
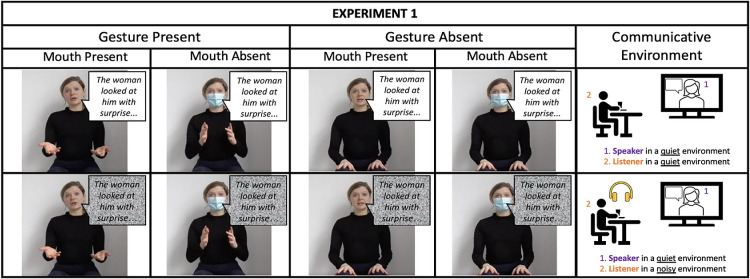
Experimental Conditions and the Communicative Environment From Experiment 1 *Note.* The yellow headphones icon indicates listening to cafeteria noise. Speaker = actress narrating stories; Listener = participant in the study. See the online article for the color version of this figure.

The stories were uttered by an actress who was a right-handed, native speaker of American English, and they were video recorded. The recordings were made with a professional camera (Zoom Q4) and took place at the actress’ house due to COVID-19 restrictions at the time of the recording. The actress was asked to tell the stories under the following conditions: (a) with her hands still on her lap and her face clearly visible, (b) with her hands still on her lap and wearing a surgical mask, (c) with spontaneous gestures and her face clearly visible, and (d) with spontaneous gestures and wearing a surgical mask. Each story was recorded in four conditions at once, rotating across conditions. As a result, there were 64 (16 Stories × 4 Conditions) different videoclips (∼1.20-min long). Next, all 64 videos were duplicated to create a degraded speech condition. That is, we extracted the audio from each videoclip and added cafeteria background noise of 0 dB signal-to-noise ratio (SNR) using a personalized matrix laboratory script (The MathWorks Inc, 2021), courtesy of Prof. Stuart Rosen. The degraded audio files were then combined with the corresponding videos resulting in 64 new videofiles with background noise. The goal of manipulating speech clarity in this way was to represent a communicative environment, in which the cues were produced by the speaker in silence but were heard/seen in noise (e.g., watching TV news in a noisy café). Altogether, there were eight experimental conditions (2 Gesture Presence × 2 Mouth Presence × 2 Speech Clarity), and each participant was exposed to each condition twice, completing 16 stories within the experiment. Figure 1 depicts experimental manipulations in Experiment 1.[Fig fig1]

#### Procedure

The experiment was created in Gorilla (https://gorilla.sc/) and lasted approximately 40 min. The task was to watch 16 videos and answer eight yes–no questions per video about the stories’ content. The order of the videos and the conditions they were presented in were randomized across participants to ensure that there was no order or learning effect. At the beginning of the experiment, participants were exposed to a practice story (in a clear speech condition with gestures and visible mouth movements) followed by eight yes–no questions appearing one after another. After each question, participants received feedback in the form of a green tick if they answered correctly or a red cross if they answered incorrectly. Feedback was provided to ensure that participants understood the task and became familiar with the different question types. At the end of the practice, participants were presented with written information reminding them that sometimes the sound would be noisy, and the speaker would wear a face mask, followed by a fragment of a video in degraded speech condition, with absent gestures and mouth movements covered with a mask as an example. The practice story was taken from the DCT practice samples and differed from the stories presented in the main experiment.

**Figure 2 fig2:**
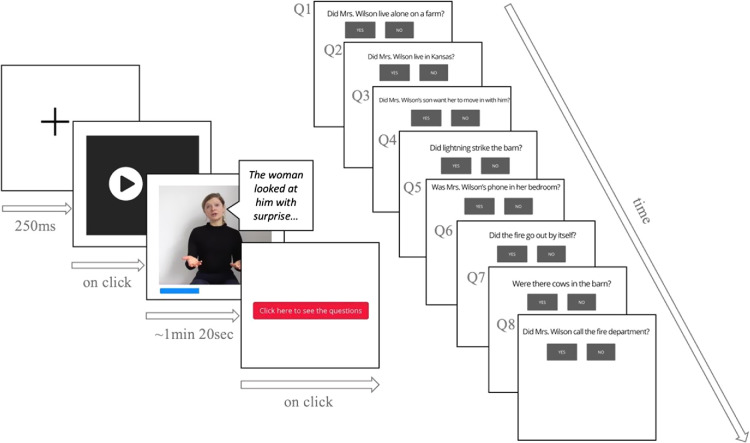
Example of an Experimental Trial With Eight Yes–No Comprehension Questions *Note*. The video depicts a speaker with visible mouth movements and producing spontaneous cospeech gestures. Q = question. See the online article for the color version of this figure.

The main trials started with a fixation cross (250 ms) followed by a video. Participants initiated the videos by clicking the “play” button. After each video, a series of eight questions appeared on the screen one by one with a fixation cross in between them (250 ms). Moving to the next question required participants to choose an answer by clicking “yes” or “no” buttons. When participants answered all eight questions, a screen with “next” button appeared, and participants could either take a short, self-timed break, or they could proceed to the next video. There was no feedback provided during the main task (see Figure 2 for an example of an experimental trial). Participants were asked to complete the experiment within an hour, and there was a progress bar indicating how far in the experiment they were. Additionally, we introduced four attention checks that appeared on random occasions within the experiment. The attention checks were yes–no questions about the videos and not related to the content of the stories (see Table 1). The *M*_accuracy score_ on those trials was 3.65/4 (*SD* = 0.60), and no participant was removed based on them.[Fig fig2]

#### Data Analysis

We carried out a logistic mixed-effect regression on the accuracy scores in R (RStudio Team, 2015) using *afex* (Singmann et al., 2016). We fitted the model with all predictors of interest, including Gesture Presence (present vs. absent), Mouth Presence (present vs. absent), Speech Clarity (clear vs. background noise), and up to the three-way interaction, following Winter (2019). Control variables of Story Type (cartoon vs. DCT) and Question Type (main idea-stated, main idea-implied, detail-stated, and detail-implied) were included in the model. All variables were categorical and were sum coded to test for main effects. The *emmeans* package (Lenth, 2022) was used to obtain estimated marginal means. We also included By-Participant and By-Story intercepts to deal with nonindependence of the data and to account for participants’ and stories’ variability. We tried fitting the maximum random structure following Barr et al. (2013) and tested the models’ fit using principal component analysis with the *RePsychLing* package (Baayen et al., 2015). We simplified the random structure based on the effect that had the least variance. To ensure convergence, *bobyqa* optimizer was used. The best fitting model contained the random slope of Speech Clarity By-Participant. The size and the direction of the effects were assessed based on the coefficients following Jaeger (2008). There was no multicollinearity (all variance inflation factors <1.6 based on *car* package; Fox & Weisberg, 2019). Before the analyses, outliers were identified and consequently removed. This included 12 trials that were answered quicker than 200 ms and three questions due to overall accuracy below 3 *SD* from the mean. For data cleaning and plotting, we used *tidyverse* (Wickham et al., 2019), *ggplot2* (Wickham et al., 2016), *sjPlot* (Lüdecke, 2022), and *beeswarm* (Eklund & Trimble, 2021) packages.

### Results and Discussion

**Figure 3 fig3:**
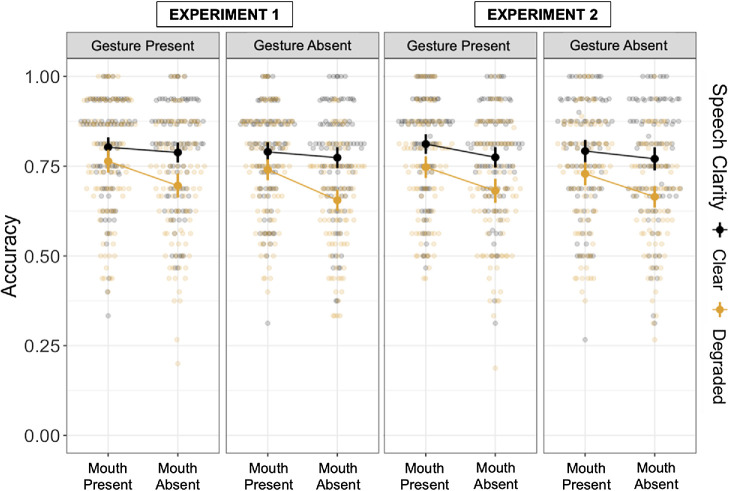
Plotted Accuracy Results From Experiments 1 (Left) and 2 (Right) *Note*. See the online article for the color version of this figure.

There was a significant main effect of Gesture Presence (*b* = 0.07, *SE* = 0.02, *z* = 2.99, *p* < .01), indicating that participants performed overall better when the gestures were present compared to when they were absent. This predictor did not interact with any other predictors. There was also a significant main effect of Mouth Presence (*b* = 0.13, *SE* = 0.02, *z* = 5.81, *p* < .001) and Speech Clarity (*b* = 0.21, *SE* = 0.02, *z* = 8.59, *p* < .001), as well as the interaction between these two predictors (*b* = −0.08, *SE* = 0.02, *z* = −3.48, *p* < .001). Follow-up Bonferroni pairwise comparison showed that comprehension was particularly hindered when the speech was degraded and mouth movements were covered by the mask, but comprehension was equally good (as in the clear condition) when the mouth movements were visible (all *p*s < .001). There was no mask effect in the clear condition (*p* > .05). The control variable of Question Type was also significant (*p*s < .001), with questions referring to main ideas being answered more accurately than questions about details and implied main ideas being answered more accurately than stated main ideas. No other effects were significant. By-Participant intercept explained 32% of additional variance, whereas By-Story intercept explained 3.5% of additional variance. Slope of Speech Clarity was <1%, suggesting a similar relationship between accuracy performance and Speech Clarity variable for all participants. Descriptive statistics are shown in Table 2 (top panel), with full results presented in Table 3 (top panel) and plotted in Figure 3 (left panel).[Table tbl2][Table tbl3][Fig fig3]

The results from Experiment 1 show that naturally occurring cospeech gestures and speech-linked mouth movements influence how listeners comprehend spoken discourse. Specifically, we found that gestures facilitate comprehension across various listening conditions, while mouth movements are beneficial only when listeners process discourse in a noisy environment. This finding aligns with the idea that gestures and mouth movements tap into different processing levels. Gestures often operate at a higher level of semantics and pragmatics due to their iconicity, which supports the construction of situation models (Feyereisen, 2006; Murgiano et al., 2021). In contrast, mouth movements operate at a lower sensorimotor level of phonology and phonetics. As such, observing mouth movements becomes particularly useful in noisy environments, as they help disambiguate speech sounds, thereby facilitating comprehension. The findings also potentially indicate a hierarchical organization of visual cues during discourse comprehension, with gestures showing greater influence than mouth movements (Hirata & Kelly, 2010). However, it is important to note that, in the current experiment, the speaker was consistently in a quiet environment, and it remains unclear whether similar effects of gestures and mouth movements would be observed if the speaker were to produce these cues in a noisy environment (Trujillo et al., 2021).

Finally, the results concerning the question type provide some novel insight into how comprehenders process discourse. We found that listeners performed better when answering questions about implied main ideas compared to those explicitly stated, in line with a predominantly inferential and heuristic view of discourse processing (Kintsch, 1998; Kintsch & van Dijk, 1978; McNamara & Magliano, 2009). Indeed, in real-life conversations, speakers frequently leave “informational gaps” (Clark & Haviland, 1977) that listeners must fill by constructing explicit or implicit inferences based on general knowledge and previous experiences to derive meaning (Graesser et al., 1994).

## Experiment 2

Experiment 2 assesses the impact of cospeech gestures, visible mouth movements, and listening conditions on discourse comprehension, when both the speaker and the listener are in the same communicative environment, that is, either in a quiet or noisy environment. Investigating the role of multimodal cues in such a communicative environment is important, as both noisy and quiet conditions are very commonly occurring ones in face-to-face communication.

### Method

#### Participants

We conducted power calculations using *mixedpower* package (Kumle & Draschkow, 2021). The analysis consisted of building a mixed-effect regression model based on the coefficients from Experiment 1 and testing power for each effect size using different sample sizes. The results indicated that recruiting 100 participants will be enough to detect the interaction between mouth movements and speech clarity with more than 90% power (at α level of .05, with 1,000 stimulations). As such, we recruited 100 native speakers of American English via Prolific (https://www.prolific.co/). As in Experiment 1, they were all right-handed monolinguals, with no language, hearing, vision, or neurological deficits. The data from four individuals were removed: One individual performed below 3 *SD* from the group mean, and three other individuals had average reaction times above 3 *SD* from the group mean. The data from the remaining 96 participants (*M*_age_ = 31.18, *SD* = 6.02; 47 females, 49 males) were used for the analyses.

#### Materials

**Figure 4 fig4:**
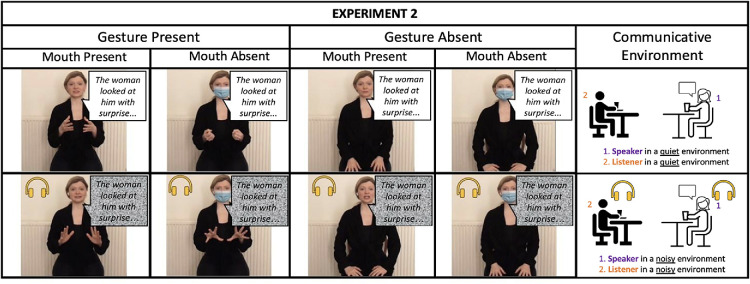
Experimental Conditions and the Communicative Environment From Experiment 2 *Note*: The yellow headphones icon indicates listening to cafeteria noise. Listener = participant in the study; speaker = actress narrating stories. See the online article for the color version of this figure.

The materials for Experiment 2 included the same 16 stories (eight DCT and eight cartoons) with eight yes–no comprehension questions per story. The stories were uttered anew by the same actress and were video recorded with a Zoom Q4 camera at her house 1 year later. All narrated stories were presented in a randomized order to ensure that the actress maintained spontaneity and naturalness in her performance. This approach also aimed to prevent the actress from memorizing the stories and/or modifying her voice/movements in a predetermined manner. The actress narrated the stories with and without gestures, as well as with and without face mask, following the conditions in Experiment 1 and resulting in a total of 64 recorded stories. Additionally, the actress reproduced all 64 stories under the same gesture and mask conditions, but this time she also wore white wireless earphones and listened to the 0 dB SNR cafeteria background noise. Implementing this procedure was hypothesized to drive the speaker to modulate her voice and the multimodal cues she produced in the noisy listening environment (eliciting a multimodal Lombard effect; e.g., Trujillo et al., 2021). Note that the background noise in the earphones is the same noise that was later used to degrade the audio for the degraded speech clarity condition (see more below). The volume level in the earphones was adjusted so that it was medium-to-very loud but did not create any discomfort to the actress. Given that participants were not informed about the actress experiencing noise through her earphones and considering the size of the earphones, we believe that this visual manipulation had no significant impact on participants’ performance. Finally, the audio from the 64 videos where the actress was wearing earphones was extracted, and the cafeteria background noise of 0 dB SNR was added, mimicking the steps introduced for Experiment 1. This was done to maintain consistency in the degraded speech condition to which participants were exposed across experiments. Participants watched all 16 stories and were exposed to each condition twice. Figure 4 depicts experimental manipulations in Experiment 2.[Fig fig4]

#### Procedure

Experiment 2 was built in Gorilla (https://gorilla.sc/) and followed the exact procedure as in Experiment 1 (see the Procedure section under Experiment 1).

#### Data Analysis

Following Experiment 1, we analyzed the data using a logistic mixed-effect regression to assess the effect of Gestures Presence, Mouth Presence, Speech Clarity, and their interactions (see the Data Analysis and the Results and Discussion sections under Experiment 1 for more details about the models). There was no multicollinearity (variance inflation factors <1.6), and outliers were removed (27 trials that were answered quicker than 200 ms and two questions due to overall accuracy below 3 *SD* from the mean).

### Results and Discussion

There was a significant main effect of Gesture Presence (*b* = 0.05, *SE* = 0.02, *z* = 2.14, *p* = .03), such that participants performed better when the gestures were present compared to when they were absent. The effect of gesture is consistent with the one found in Experiment 1, however, with a smaller effect size. There was also a significant main effect of Mouth Presence (*b* = 0.12, *SE* = 0.02, *z* = 5.15, *p* < .001) and Speech Clarity (*b* = 0.25, *SE* = 0.03, *z* = 8.65, *p* < .001), as well as a marginal interaction between the two (*b* = −0.04, *SE* = 0.02, *z* = −1.71, *p* = .09). Considering the main effects observed for Mouth Presence and Speech Clarity, which indicate that observing mouth movements is beneficial for listeners and that degraded speech is more challenging than clear speech, the marginal interaction further suggests that mouth movements are especially advantageous when speech is degraded as opposed to when it is clear (*p* < .001; Bonferroni pairwise comparison). This finding aligns with the significant interaction identified in Experiment 1. Finally, like in Experiment 1, the control variable of Question Type was also significant (*p*s < .001).^2^ Questions referring to main ideas were answered more accurately than questions about details, and implied main ideas were answered more accurately than stated main ideas. Participant intercept explained almost 30% of additional variance, and Story intercept explained almost 4%. Speech Clarity slope effect did not substantially differ between participants, although it explained more variance than in Experiment 1 (2.7%). No other effects were significant. Descriptive statistics are shown in Table 2 (bottom panel), with full results presented in Table 3 (bottom panel) and plotted in Figure 3 (right panel).

Experiment 2 successfully replicated the results of Experiment 1 for the clear condition and demonstrated similar effects for the degraded condition with new participants and rerecorded video materials. We found that observing gestures is beneficial across noisy and quiet listening conditions, and observing mouth movements is (marginally) more important in the noisy condition. Overall, these findings support the hypothesis that visual cues are weighted differently during discourse comprehension. As a reminder, the main difference between the experiments was that the speaker was situated in the same communicative environment as the listener in Experiment 2, but the speaker was always in a quiet environment in Experiment 1. This manipulation did not seem to affect the way visual cues impact discourse comprehension, as demonstrated by similar effect sizes (*b* coefficients in Experiment 1 for Gesture Presence = 0.07, Mouth Presence × Speech Clarity = −0.08; *b* coefficients in Experiment 2 for Gesture Presence = 0.05, Mouth Presence × Speech Clarity = −0.04).^3^ Finally, Experiment 2 also replicated the finding that comprehenders make inferences while processing discourse, as questions about implied main ideas were processed more accurately than stated ones.

## Follow-Up Analyses

Across the experiments, we observed a robust, albeit small, effect of gestures on discourse comprehension. Since gestures were unscripted in our study, the speaker had the freedom to produce different types of gestures to different extents, and it is not clear whether specific gestures (e.g., representational) may drive this effect. Thus, we conducted follow-up analyses to investigate the impact of gesture type, frequency, and amplitude on discourse comprehension.

### Gesture Type and Frequency Analysis

The analysis consisted of annotating all gestures in the videos (gesture present condition only) into one of three types: (a) representational and deictic gestures (including iconic and metaphoric gestures, abstract and concrete points, as well as emblems, i.e., conventionalized gestures such as thumbs up to represent an agreement) to describe their meaningful nature, (b) beat gestures, and (c) pragmatic gestures, following the lab coding manual (available upon request). Comparable analyses were conducted by Zhang, Frassinelli, et al. (2021), where they investigated the influence of spontaneously produced iconic, deictic, and beat gestures on discourse comprehension in neurotypical adults. We then tested a logistic mixed-effect regression model with gesture types (Representational/Deictic Gestures, Beat Gestures, and Pragmatic Gestures; all centered on the mean) as predictors, Story Type as a control variable, accuracy as the dependent variable, and random intercepts of Participant and Story. Only trials when the gestures were present were tested.

**Figure 5 fig5:**
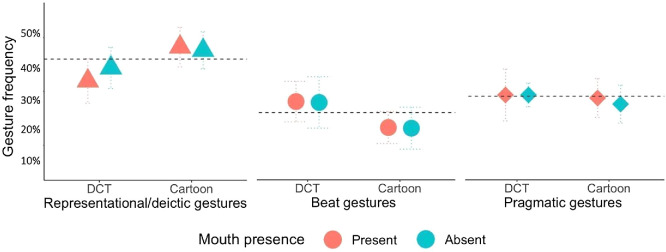
Results of the Gesture Type and Frequency Analysis *Note*. The *x*-axis represents gesture type (representational/deictic gestures, beat gestures, pragmatic gestures) averaged by story type (DCT, cartoon), and the *y*-axis represents gesture frequency (percentage). Color refers to the mouth presence condition (red = present, turquoise = absent). Error bars are standard deviations from the mean. DCT = Discourse Comprehension Test. See the online article for the color version of this figure.

AK annotated all the gestures in Experiment 1 using EUDICO Linguistic Annotator (Version 6.3, 2022). Coding reliability (10% of the total videos) between AK and a student assistant showed high intraclass correlation (Cronbach’s α > .9). The actress produced on average 59 (*SD* = 6.14) and 60 (*SD* = 5.24) gestures per video when mouth movements were present versus absent, respectively. Most of the gestures were representational/deictic (43%), followed by pragmatic (32%), and beat gestures (25%). Numerically, there were more representational/deictic gestures produced during cartoon than DCT stories, and more beats during DCT than cartoon stories (see Figure 5). A greater frequency of representational/deictic gestures in cartoons compared to DCT was expected, given the inherently action-oriented nature of cartoons. Conversely, DCT stories, being more abstract, were expected to involve fewer instances of these gestures. The statistical analysis showed no significant effects on accuracy performance (see Table 4); thus, gesture coding was not carried out for Experiment 2.[Fig fig5][Table tbl4]

### Gesture Amplitude Analysis

The goal of the gesture amplitude analysis was to provide initial assessment of whether the size of gestures produced in Experiment 1 differed from those produced in Experiment 2. Similar analyses were run in, for example, Trujillo et al. (2021) to assess the Lombard effect. We randomly selected one of the stories from our stimuli and analyzed the speaker’s kinematics produced in silence (from a video from Experiment 1) and in noise (from a video from Experiment 2). The analysis consisted of an automatic 2D key point detection of movements of arms and hands using OpenPose (Cao et al., 2019). That is, we generated *x* and *y* coordinates for six of the body parts: right and left shoulders, elbows, and wrists. We then calculated the distance between the coordinates for each sentence (14 sentences per story) and for each frame. Larger distance indicates bigger movements. Finally, for each sentence, we took the average distance value for each key point, scaled it, and used it as our dependent variable in a linear mixed-effect regression analysis (with the *lme4* package; Bates et al., 2015). The predictors included: Key Point (with six levels, corresponding to the *x* and *y* coordinates for the six body parts), Experiment (Experiment 1 vs. 2), and the interaction between the two. We also entered Sentence as a random intercept. The categorical variables were dummy coded.

**Figure 6 fig6:**
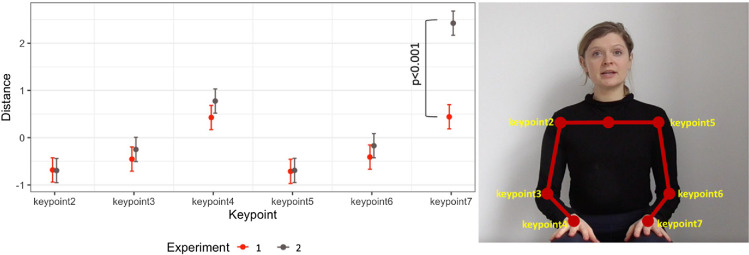
Results of the Gesture-Amplitude Analysis *Note*. The left panel: The *x*-axis represents different key points used in the analysis, and the *y*-axis is the scaled distance. Colors represent experiment type. Error bars are standard errors of the mean. The right panel shows the schematic representation of key points annotation. DCT = Discourse Comprehension Test. See the online article for the color version of this figure.

The results of the Gesture Amplitude analysis showed a significant effect of Key Points 4 (right wrist; *b* = 1.11, *SE* = 0.17, *t* = 6.38, *p* < .001) and 7 (left wrist; *b* = 1.13, *SE* = 0.17, *t* = 6.46, *p* < .001) with larger distance relative to other key points. Crucially, there was also a significant interaction between Key Point and Experiment (*b* = 1.99, *SE* = 0.25, *t* = 8.09, *p* < .001), with larger distance for Key Point 7 (left wrist) in Experiment 2 than Experiment 1. Overall, these results showed that the speaker produced bigger movements (using her left hand) when in noise, suggesting that she made gestural adjustments to deal with challenging listening conditions. More broadly, these post hoc analyses provided initial evidence for the multimodal Lombard effect (Trujillo et al., 2021). Figure 6 depicts the findings.[Fig fig6]

These follow-up analyses yield two key findings. First, the speaker produced a variety of gestures while narrating stories, but the type and frequency of these gestures did not significantly impact comprehension scores. Second, the speaker used her nondominant hand more often when producing the stories in noise. This finding is in line with the multimodal Lombard effect (Trujillo et al., 2021).

## General Discussion

The present study is the first to investigate the benefit of multimodal cues, including gestures and mouth movements, for auditory discourse comprehension under clear and challenging (with background noise) listening conditions. We presented videos of a speaker who, in half of the instances, spontaneously gestured and wore a health mask that covered communicative facial cues while narrating stories. This design aimed to resemble everyday communicative settings. We investigated the impact of interactions between visual cues and listening conditions on listeners’ comprehension. Additionally, we looked at whether any potential multimodal benefit to comprehension depends on the communicative environment.

Across two experiments, we found that comprehending discourse is easier when spontaneous cospeech gestures accompany speech. This effect was small but robust and did not depend on gesture frequency/type, speech clarity, or mouth presence. We also showed that mouth movements support discourse comprehension. This effect, however, was most prominent in challenging listening conditions. Finally, we demonstrated that the multimodal benefit to comprehension was similar regardless of the communicative environment the speaker was situated in.

### Multimodal Cues Matter to Listeners (but to a Different Extent)

Multimodal cues, such as gestures and mouth movements, are part and parcel of face-to-face communication. Our findings support this view by demonstrating that these cues matter to listeners: They improve discourse comprehension, but they do so to a different extent.

Spontaneously produced cospeech gestures benefit discourse comprehension regardless of the presence of other cues or the clarity of the speech signal. This finding was observed in Experiment 1 and was then demonstrated in Experiment 2. It is also in line with previous studies on discourse comprehension showing beneficial (albeit small to medium) effect of gestures (for a meta-analysis see Dargue et al., 2019). Such gestural benefit to comprehension has primarily been attributed to typical iconic (Dargue & Sweller, 2018, 2020; McKern et al., 2021) and deictic (Goodrich Smith & Hudson Kam, 2012; Gunter et al., 2015; Sekine & Kita, 2015, 2017) gestures, which were also the most frequently produced types of gestures in this study. These gestures are known for their role in supporting the creation of situation models (e.g., Clark, 1996; Cutica & Bucciarelli, 2013; McNeill, 1992) by engaging prior perceptual–motor experiences to simulate embodied events (e.g., Glenberg & Gallese, 2012; Glenberg et al., 2013; Zwaan, 2014), and the more informative they are, the more benefit to comprehension there is (Dargue & Sweller, 2018; Krason et al., 2021; Zhang, Frassinelli, et al., 2021).

In this study, other types of cospeech gestures were also present, and their role in supporting linguistic processing has been noted, albeit differently than representational or deictic gestures. For example, pragmatic gestures—the second most common type of gestures here—may have facilitated discourse processing by maintaining an engaging and interactive character of the narratives (Bavelas et al., 1992, 1995) or by marking specific structures or functions of discourse (e.g., indicating implied information or intensifying an argument; Kendon, 2004). The benefit of beat gestures—the least frequent gestures (although note that there were more beat gestures in the DCT stories than in the cartoon stories)—to comprehension is still debatable. Some have argued that they impact auditory comprehension to a lesser extent than representational gestures (Feyereisen, 2006; Macoun & Sweller, 2016; Zhang, Frassinelli, et al., 2021), and others have suggested that beat gestures might only be useful in processing the word to which they are time-locked to rather than any other information around (Igualada et al., 2017). Here, we have demonstrated that unscripted and spontaneously produced cospeech gestures collectively contribute to discourse comprehension benefit.

While we did not find evidence that the frequency, type, and amplitude of gestures differentially impacted discourse comprehension, future studies could focus on better quantifying the informativeness of various gestures in relation to discourse comprehension. Researchers could also investigate the influence of gestures produced by different individuals to better understand how gesture idiosyncrasy impacts comprehension.

Although some studies have shown that cospeech (iconic) gestures are particularly helpful in challenging listening conditions (Drijvers & Özyürek, 2017; Holle et al., 2010; Krason et al., 2021; Obermeier et al., 2012), our results demonstrate that listeners always take gestures into account during face-to-face discourse even when listening conditions are clear. We propose that the different results can be explained in terms of task difficulty. That is, performance on word/gesture comprehension tasks (e.g., free recall, Drijvers & Özyürek, 2017; picture verification, Krason et al., 2021; or assessing congruency between gesture and homonymous words, Holle et al., 2010) is most often at ceiling when the speech is clear, so the effect of gestures could only be observed when the task is more challenging (i.e., when the speech was degraded). This is not the case in our discourse comprehension task; thus, we observe the effect of gestures in both clear and degraded conditions.

Additional evidence comes from recent studies by McKern et al. (2021) and Wilms et al. (2022), who showed that iconic gestures benefit narrative recall and sentence recognition, respectively, regardless of the noise. These findings, along with ours, jointly support the speech–gesture integration account according to which cospeech gestures are automatically and obligatorily processed alongside speech (e.g., Kelly et al., 2010; McNeill et al., 1994). Zhang, Frassinelli, et al. (2021) further showed that representational and beat gestures (as well as prosody and mouth movements) modulate the incoming linguistic information in naturalistic settings, and our study extends their finding by demonstrating that these modulations lead to quantifiable improvements in discourse comprehension.

In contrast to cospeech gestures, mouth movements are only used when speech is challenging. This effect was consistent across experiments and is well-established in the literature (e.g., Ma et al., 2009; L. A. Ross et al., 2007; Sumby & Pollack, 1954). Studies have suggested that seeing a speaker’s mouth movements supports phonological processing and constrains lexical competition, which is crucial in adverse listening conditions when the auditory channel provides only suboptimal acoustic information for phonological processing during comprehension (for a review, see Peelle & Sommers, 2015). Our study has further demonstrated that the audiovisual speech enhancement in challenging listening conditions holds even with naturalistic manipulation of mouth presence, that is, when a speaker wears a surgical mask. In contrast, a similar close coupling between listening conditions and gestures is less probable, as gesture primarily supports semantic and pragmatic processing levels.

A number of recent studies have investigated the effect of masks on speech recognition. Toscano and Toscano (2021) examined the impact of surgical mask, two different cloth masks, and an N95 respirator on auditory-only sentence recognition under different noise levels. They found that wearing a mask had little impact on the accuracy performance under low-level noise, but it mattered, and depended on a mask type, under high-level noise. Interestingly, the surgical mask was the only type that did not show an effect under any noise conditions. Building on these findings, V. A. Brown et al. (2021) further demonstrated that wearing a mask (regardless of its type) makes sentence comprehension in noise more challenging than when mouth movements are visible. Here, we showed a similar mask effect for discourse comprehension. Given that Toscano and Toscano (2021) found that the use of surgical masks has no impact on auditory processing of speech in noise, the effect of mask found in our study can be attributed to the fact that the mask obscured information from visual speech rather than muffled spoken signal.

The finding that mouth movements are primarily helpful when speech is degraded but gestures benefit comprehension across different speech clarity conditions suggests that these visual cues are flexibly weighted by listeners (Krason et al., 2021; Zhang, Frassinelli, et al., 2021) because they support linguistic processes differently (Hirata & Kelly, 2010). That is, whereas mouth movements support temporal and phonological encoding of the incoming speech, cospeech gestures support processing of high-level information (e.g., semantic encoding), and listeners will use a cue or a combination of cues that is the most informative in a given context (Krason et al., 2021; Zhang, Frassinelli, et al., 2021). Some researchers have also suggested a “double enhancement” effect, that is, greater benefit to spoken word comprehension when both gesture and mouth cues are present, particularly in challenging listening conditions (Drijvers & Özyürek, 2017). Here, we showed that when cospeech gestures convey sufficient information to correctly interpret spoken passages, mouth movements become less important.

Finally, we also contrasted two possible communicative environments in which cues can be produced and perceived. One scenario assumes that a speaker produces cues in a quiet environment, but listeners perceive them in noise (e.g., watching TV news in a noisy café; Experiment 1), and the other scenario depicts a situation where a speaker produces cues in noise and listeners also perceive them in noise (e.g., having a conversation in a noisy café; see Experiment 2). Such manipulation was introduced to investigate whether the multimodal benefit depends on the listening conditions in which the cues are produced. Although we found that the speaker produced bigger movements with her left (nondominant) hand in Experiment 2 than in Experiment 1, we did not find evidence that this gestural adjustment had a significant effect on comprehension (see Footnote 3 and effect sizes). Larger left-hand movements potentially suggest that the speaker was trying to compensate for the noise disruption (which is in line with the multimodal Lombard effect; Trujillo et al., 2021). However, since the speaker did not engage in direct interaction with the listener (participants), it remains unclear to what extent this absence of interaction may account for the observed lack of additional benefit to comprehension. For instance, in studies such as Trujillo et al. (2021) and Garnier et al. (2018), listeners actively participated in a conversation with the speaker, potentially enhancing multimodal modulations and their dynamic weighting. It is also possible that the relatively small size of the videos may have prevented participants from noticing the difference in gesture amplitude, resulting in null findings.

### Advancing Naturalistic Language Comprehension Models

The work presented in this article is a building block in the development of naturalistic language comprehension models (Hasson et al., 2018; Nastase et al., 2021; Skipper, 2015; Vigliocco et al., 2024). It advances our understanding of discourse processing by extending findings on the benefits of visual cues, including various types of gestures, such as representational, deictic, beat, and pragmatic gestures, as well as mouth movements, to (semi) naturalistic comprehension. We challenged the assumption that gestures are extraneous rather than central to language comprehension by demonstrating that listeners consistently use the information from gestures during discourse comprehension, even in clear listening conditions. Our findings also align with the notion that cospeech gestures often modulate the semantic or pragmatic level of processing, while mouth movements tap onto phonetics/phonology, providing insights into how and when gestures and mouth movements support discourse processing. Specifically, listeners use gestures across various listening conditions to support the creation of situation models, and they use mouth movements to disambiguate speech in challenging listening conditions.

Studying interactions between communicative cues during discourse processing poses several challenges. There are large individual differences in how much people benefit from gestures and mouth movements, and it is unclear if similar multimodal benefits to discourse would be found for other populations. For example, individuals with poorer memory abilities extract less communicative information from gestures (McKern et al., 2021; Schubotz et al., 2020), and second language learners tend to benefit less than native speakers (Drijvers & Özyürek, 2020; Zhang et al., 2023). Similar findings were observed for people with poststroke aphasia, who, despite benefitting from visible mouth movements, showed a smaller effect than age-matched individuals without aphasia (Krason et al., 2023). Moreover, cospeech gestures and mouth movements are not the only communicative cues during face-to-face interactions. Studies have shown that, for instance, word predictability and prosody similarly modulate brain activity during naturalistic discourse comprehension (Zhang, Frassinelli, et al., 2021). Researchers have also demonstrated a strong link between other cues, such as beat gestures and prosody (Esteve-Gibert & Prieto, 2013), pragmatic gestures and negation movements (e.g., headshake; Kendon, 2004), eye gaze and cospeech gestures (Gullberg & Holmqvist, 1999), as well as between nodding and blinking (Hömke et al., 2018). All these cues are integral to face-to-face communication, and situating language in a physical and communicative environment is crucial for a thorough understanding of language processing *in situ* (Holler, 2022; Holler & Levinson, 2019; Levinson & Holler, 2014; Reggin et al., 2023; Murgiano et al., 2021). The present study supports this view.

## Supplementary Material

10.1037/xlm0001399.supp

## Figures and Tables

**Table 1 tbl1:** Comparison Between the Selected DCT (Brookshire & Nicholas, 1993) and Cartoon Stories (Based on Episodes From Tom and Jerry and Sylvester and Tweety Pie)

Story type	DCT (*n* = 8)	Cartoon (*n* = 8)
Example stories	“Neil Williams was short of money. The new term was about to begin and he didn’t have enough money to pay his tuition. So, 1 day, he walked to his parents’ home and borrowed their car. Then he drove to the bank to get a student loan. The loan officer at the bank was a tough old woman who always said she had never made a bad loan. …”	“One afternoon last summer, Sylvester had a clever plan to catch Tweety Pie. He hid in a room in a building of ‘Bird Watchers’ Society’, which was just across the street from Tweety Pie’s apartment. Tweety Pie was happily swinging in his cage on the windowsill. Sylvester looked out from the window and used binoculars to get a better view on Tweety Pie’s apartment. In less than 2 min, he spotted Tweety Pie’s cage …”
Example questions	1 Was Neil a high school student? (No; MI-I) 2 Did Neil’s parents live nearby? (Yes; DT-I) 3 Did Neil go to the bank to get a loan? (Yes; MI-S) 4 Did Neil need the money to start a new business? (No; MI-S) 5 Did Neil own a car? (Yes; DT-S) 6 Did Neil go to the bank in the morning? (No; DT-I) 7 Did Neil tell the woman that he had a cheese sandwich for lunch? (No; DT-S) 8 Did Neil get the loan? (Yes; MI-I)	1 Was Sylvester hiding in the same building where Tweety Pie? (No; DT-S) 2 Was Tweety Pie outside the building? (No; DT-I) 3 Did Sylvester spot Tweety Pie’s cage through binoculars? (Yes; MI-S) 4 Was Tweety Pie scared of Sylvester? (Yes; DT-I) 5 Did Sylvester use the main door to enter the Tweety Pie’s building? (Yes; DT-S) 6 Did Sylvester follow all the signs of Tweety Pie’s building? (No; MI-I) 7 Was Sylvester escorted out of the building? (No; MI-S) 8 Did Sylvester have a headache at the end? (Yes; MI-I)
Example attention check questions	1 Was the actress wearing a black top?	1 Was the actress standing?
Average number of words (all stories = 199.8)	203.3	196.3
Average number of unfamiliar words (all stories = 2.7)	2.4	3
Average number of sentences (all stories = 13.7)	13.9	13.5
Sentence length (all stories = 14.6)	14.7	14.6
Average number of subordinate clauses (all stories = 9.9)	9.3	10.5
Listening difficulty (all stories = 4.6)	4.6	4.7
*Note*. DCT = Discourse Comprehension Test; MI-I = main idea-implied; DT-I = detail-implied; DT-S = stated details; MI-S = main idea-stated.		

**Table 2 tbl2:** Descriptive Statistics (Proportion Correct and Standard Deviations in Brackets) From Experiments 1 and 2

Clear speech				Degraded speech			
Gesture present		Gesture absent		Gesture present		Gesture absent	
Mouth present	Mouth absent	Mouth present	Mouth absent	Mouth present	Mouth absent	Mouth present	Mouth absent
Experiment 1 (*n* = 98)							
0.80 (0.40)	0.79 (0.41)	0.79 (0.41)	0.77 (0.42)	0.76 (0.43)	0.70 (0.46)	0.74 (0.44)	0.66 (0.48)
Experiment 2 (*n* = 96)							
0.81 (0.40)	0.78 (0.42)	0.79 (0.41)	0.77 (0.42)	0.74 (0.44)	0.69 (0.47)	0.73 (0.45)	0.67 (0.47)

**Table 3 tbl3:** Results of the Mixed-Effects Logistic Regression Models for Experiment 1 (Top) and Experiment 2 (Bottom)

Experiment 1				
Random effect	Variance	*SD*		
Participant(Intercept)	0.32	0.57		
SpeechClarity1	0.01	0.09		
Story(Intercept)	0.04	0.19		

Fixed effect	*b*	*SE*	*z*	*p*
(Intercept)	1.23	0.08	15.77	<.001***
GesturePresence1	0.07	0.02	2.99	<.01**
MouthPresence1	0.13	0.02	5.81	<.001***
SpeechClarity1	0.21	0.02	8.59	<.001***
GesturePresence1:MouthPresence1	−0.01	0.02	−0.44	.66
GesturePresence1:SpeechClarity1	−0.02	0.02	−0.80	.42
MouthPresence1:SpeechClarity1	−0.08	0.02	−3.48	<.001***
GesturePresence1:MouthPresence1:SpeechClarity1	0.01	0.02	0.27	.79
Control variable	*b*	*SE*	*z*	*p*
StoryType1	0.02	0.05	0.45	.65
QuestionType1	0.44	0.04	10.71	<.001***
QuestionType2	−0.29	0.04	−7.90	<.001***
QuestionType3	−0.30	0.04	−8.41	<.001***

Experiment 2				
Random effect	Variance	*SD*		
Participant(Intercept)	0.30	0.54		
SpeechClarity1	0.03	0.16		
Story(Intercept)	0.04	0.20		

Fixed effect	*b*	*SE*	*z*	*p*
(Intercept)	1.19	0.08	15.17	<.001***
GesturePresence1	0.05	0.02	2.14	.03*
MouthPresence1	0.12	0.02	5.15	<.001***
SpeechClarity1	0.25	0.03	8.65	<.001***
GesturePresence1:MouthPresence1	0.01	0.02	0.26	.80
GesturePresence1:SpeechClarity1	−0.01	0.02	−0.45	.65
MouthPresence1:SpeechClarity1	−0.04	0.02	−1.71	.09
GesturePresence1:MouthPresence1:SpeechClarity1	0.01	0.02	0.24	.81
Control variable	*b*	*SE*	*z*	*p*
StoryType1	0.02	0.05	0.44	.66
QuestionType1	0.42	0.04	10.22	<.001***
QuestionType2	−0.25	0.04	−6.47	<.001***
QuestionType3	−0.29	0.04	−7.88	<.001***
*Note.* Sum contrasts: GesturePresence1 = “Present”, MouthPresence1 = “Present”, SpeechClarity1 = “Clear”, StoryType1 = “DCT”, QuestionType1 = “MI-I”, QuestionType2 = “DT-I”, QuestionType3 = “DT-S”. *SE* = standard error; DCT = Discourse Comprehension Test; MI-I = main idea-implied; DT-I = detail-implied; DT-S = stated details.				
* *p* < .05. ** *p* < .01. *** *p* < .001.				

**Table 4 tbl4:** Results of the Gesture Type and Frequency Analysis

Fixed effect	*b*	*SE*	*z*	*p*
Intercept	1.27	0.07	17.44	<.001
MeaningfulGestures_z	0.01	0.05	0.22	.83
BeatGestures_z	−0.02	0.05	−0.38	.70
PragmaticGestures_z	0.06	0.05	1.28	.20
StoryType1	0.00	0.06	−0.07	.94
*Note*. *SE* = standard error.				

## Footnotes

1 Note the difference in labeling the experiments here (Experiment 1 vs. Experiment 2) and in the preregistrations (Experiment 1a vs. Experiment 1b).

2 We also tested models with Question Type in interactions with Gesture Presence, Mouth Presence, and Speech Clarity to further investigate the effect of Question Type on accuracy performance. Only the interaction between Mouth Presence1:QuestionType3 in Experiment 1 was significant (*p* = .02), such that participants performed better on questions about stated details compared to the average effect of all question types when mouth movements were present than absent. Full results from these models can be found on the OSF at https://osf.io/6zxuw/.

3 We also tested a logistic mixed-effect model with aggregated data from both experiments and an additional predictor of Experiment (Experiment 1 vs. 2). The model showed a nonsignificant effect of Experiment (*b* = 0.02, *SE* = 0.04, *z* = 0.40, *p* = .69), providing little evidence that observing visual cues produced by a speaker-in-silence (Experiment 1) versus in-noise (Experiment 2) impacted comprehension differently. Full results are presented on the OSF at https://osf.io/6zxuw/.
